# An Unexpected Case of Perioperative Recurrent Bronchospasm: A Case Report and Review of the Literature

**DOI:** 10.7759/cureus.109843

**Published:** 2026-05-28

**Authors:** Zacharry Saitowitz

**Affiliations:** 1 Emergency Department, Ipswich Hospital, Ipswich, AUS

**Keywords:** airway, bronchospasm, emergency, management, perioperative

## Abstract

Bronchospasm is a condition commonly encountered in the perioperative setting and represents an airway emergency that requires immediate identification and management. While multiple factors increase the risk for bronchospasm, it can still appear in patients who are otherwise well. Of note, once treatment has been administered, it is important to continue monitoring the patient after the event to ensure full resolution. A 58-year-old medically well woman presented for an emergent laparoscopic cholecystectomy. Soon after intubation, she developed a sudden oxygen desaturation with hypotension and notable bilateral wheeze on auscultation. A rapid assessment was performed, considering issues with the endotracheal tube, airway obstruction, her depth of anaesthesia, bronchospasm, and anaphylaxis. Following this, she was treated for bronchospasm and supported briefly with metaraminol, with improvement in her vital signs and subsequent uneventful extubation. However, while in recovery, she had another episode of oxygen desaturation with a normal chest radiograph and a blood gas analysis suggesting a high A-a gradient, likely a further reactive airways event that required further treatment with bronchodilators and led to an overnight admission to the high-dependency unit for close monitoring and resolution of her hypoxia. A follow-up procedure in the next few days was pre-treated with nebulised bronchodilators, with an uneventful procedure and subsequent discharge home. A review of the literature shows the differential for bronchospasm remains broad, and multiple factors must be considered while ensuring patient safety. While treatment may be similar, it is paramount to ensure full resolution of symptoms prior to any consideration of discharge, as bronchospasm may recur in the short term with poor outcomes if not promptly addressed.

## Introduction

Bronchospasm is a condition that occurs when the bronchial muscles contract, leading to a reduction of airway calibre and reduced airflow. This may present as a sudden rise in ventilation pressures, oxygen desaturation, changes in capnography, or auscultation abnormalities such as wheezing or a silent chest. A broad differential needs to be considered when any of these signs are observed, including anaphylaxis, airway obstruction, endotracheal tube (ETT) displacement, pneumothorax, or inadequate depth of anaesthesia [1]. While bronchospasm often occurs in isolation, it can also be the result of a larger pathological process, such as the third spacing of fluid in anaphylaxis or acute heart failure from a large pulmonary embolism. Bronchospasm can also be imitated with findings from other differentials, such as the turbulent and reduced airflow from a kinked ETT or a mucus plug. This diagnostic challenge highlights the importance of a rapid approach to exclude bronchospasm caused by airway irritation versus as part of other pathologies. Management of isolated bronchospasm differs from that of bronchospasm associated with broader pathology, as the underlying condition necessitates different therapies. Several mechanisms underlie the pathophysiology of bronchospasm, with risk factors including smoking history, atopy, and recent respiratory infections [2-4]. The decrease in airflow and alveolar ventilation constitutes an anaesthetic emergency requiring swift recognition and intervention. Importantly, bronchospasm shares features with other pathologies that must also be addressed and excluded to ensure patient safety [3,4]. While bronchospasm typically results from airway irritants affecting sensitive or reactive airways, it has also been reported during all phases of a general anaesthetic, including recovery, when most irritants are unlikely to be present [1]. This is the case of a medically well woman experiencing multiple periprocedural bronchospasm events, emphasising the importance of maintaining a broad differential during crisis management.

## Case presentation

A 58-year-old woman presented to our hospital for an emergent laparoscopic cholecystectomy. Her background includes smoking, but not asthma or chronic airway disease and no history of inhaler use and was deemed an American Society of Anesthesiologists class 2E. She had previous anaesthetics without incident. Induction was performed with 200 mcg fentanyl, 50 mg rocuronium, and propofol given at 6 mcg/mL with a Marsh model target-controlled infusion (TCI). A size 7 ETT was placed and confirmed with a direct view of the placement, capnography, and bilateral chest wall rise. About 10 minutes post-induction, the patient’s oxygen saturation dropped from 98% to 88% with a rapid increase in peak pressures from around 20 to 30-35 cmH2O, as well as concurrent capnography consistent with bronchospasm. Blood pressure (BP) dropped from around 150/70 mmHg to 90/40 mmHg during the same time frame. The heart rate was initially stable at around 60 beats per minute, but suddenly increased to about 110 beats per minute at this time. ETT depth was checked and confirmed to have remained in its original depth, ruling out tube displacement. The chest was noted to have good bilateral rise and fall, making a large pneumothorax or lung collapse less likely. Auscultation revealed bilateral wheeze without coarse crackles, arguing against acute pulmonary oedema or a large aspiration event. There were no obvious skin changes or sudden rash noted on the patient, which would have been more concerning for anaphylaxis; however, the absence of this does not rule out anaphylaxis. The patient was given 800 mcg of inhaled salbutamol, 8 mg IV dexamethasone, and a 10 mmol IV magnesium infusion, and sevoflurane was commenced at 0.7 minimum alveolar concentration (MAC) in quick succession with a return to 98-100% oxygen saturations over the next four minutes. A metaraminol infusion was started after the above treatments to ensure no further BP drops. At this stage, she was given 2 g of IV cefazolin. After 10 minutes, her BP had improved to 100/80 mmHg, and the metaraminol infusion was ceased at the next five-minute check as her BP had shot up to 180/90 mmHg. Propofol had been decreased to 3.5 mcg/mL via the TCI following induction, but at this stage increased to 4 mcg/mL to deepen her anaesthesia. The remainder of the case was uneventful, and she was successfully extubated. Neither a venous/arterial blood gas nor a tryptase level was sent during her time in theatre. Due to her response to salbutamol, the main differential was that this event was most likely due to bronchospasm without significant concern for other aetiologies. As mentioned above, tube displacement was effectively ruled out, and with equal bilateral chest rise and fall, it was unlikely that a tube obstruction had occurred; however, this remains a possibility. Additionally, the equal rise and fall would suggest against a massive pneumothorax. Anaphylaxis would unlikely have responded to those initial management strategies. Ongoing hypotension resistant to metaraminol or difficulty with ventilation despite the salbutamol and deepening of anaesthesia would have prompted greater concern and management for anaphylaxis. 

While in recovery, she experienced another oxygen desaturation, decreasing to the low 80% range, despite using high-flow nasal prongs. She was switched to a 6L Hudson mask for nebulised salbutamol and ipratropium. The patient did not show physical signs of respiratory distress, despite the measured hypoxemia. Respiratory rate remained between 10 and 16 breaths/min with bilateral expiratory wheeze with some quiet zones. A chest radiograph obtained at the time showed no acute pathology, ruling out significant atelectasis, consolidation, or pneumothorax. An arterial blood gas sample obtained while receiving the nebulised medications showed a pH of 7.30, PaCO2 of 53 mmHg, PaO2 of 70 mmHg, and a bicarbonate of 25 mmol/L on an FiO2 of 40% with saturations of 90% on peripheral monitoring at the time. Due to the previous intraoperative episode responding well to bronchodilators without the need for adrenaline, and stable BP in recovery during this episode, the ongoing hypothesis was that she was having recurrent bronchospasm. She was admitted to the high-dependency unit with a gradual reduction in respiratory support overnight and stepped down to the ward the following day. This patient required an additional endoscopic retrograde cholangiopancreatography a few days later, for which she received a dose of nebulised salbutamol and ipratropium an hour before the subsequent uneventful procedure. She was discharged home the following day. The decision for only bronchodilator prophylaxis prior to her uneventful next anaesthetic gives more evidence towards bronchospasm as the isolated pathology, but further testing would have aided the exclusion of other pathologies. While it remains the most likely diagnosis, no plans for further allergy testing or anaesthetic clinics were made. 

## Discussion

Bronchospasm occurs when the smooth muscle surrounding the conducting airways contracts due to some irritant, including as part of a larger anaphylaxis reaction. It is more common in patients with a history of reactive airways disease, such as asthma or chronic obstructive pulmonary disease (COPD), smoking, and obesity [1-4]. Bronchospasm can cause sudden ventilation failure, and while it should be considered, especially in ventilated patients, a broad differential diagnosis must be initially considered, such as tube kinking, mucus plugging, cuff overinflation, or anaphylaxis [3,4]. The incidence of bronchospasm has been reported to be 1.3-1.7 per 1000 patients undergoing general anaesthesia, while anaphylaxis is much rarer at rates of one in 5,000-10,000; however, it must still be considered, particularly when bronchospasm is accompanied by hypotension [5-7]. Table 1 lists some signs and differentials to consider.

**Table 1:** Signs suggestive of bronchospasm and alternative diagnoses to consider ETT: endotracheal tube

Signs of bronchospasm	Differentials to consider
Increased ventilatory pressures	Anaphylaxis
Desaturation	ETT displacement or endobronchial intubation
Prolonged expiratory phase	Kinking or obstruction of the tube
Wheeze (silent chest in severe cases)	Airway irritation/secretions/soiling
Rising end-tidal CO_2_	Laryngospasm
Reduction in tidal volumes	Pneumothorax
	Inadequate depth of anaesthesia
	Pulmonary embolism

In normal physiology, airways have neuronal, biochemical, and inflammatory factors that affect ventilation. Airway contraction is controlled by the autonomic nervous system, specifically vagal nerve impulses that ultimately release acetylcholine, which acts as a ligand for muscarinic receptors on bronchiolar smooth muscle [2,8]. This then results in increased intracellular calcium concentrations, which cause contraction [8]. Additionally, beta-2 adrenergic receptors are found throughout the bronchial smooth muscle, which, when activated with an agonist, increase cyclic adenosine monophosphate and decrease intracellular calcium, resulting in smooth muscle relaxation. This becomes increasingly important with the growing number of people on beta-blocker medications, although beta-1-selective antagonists, such as esmolol, appear to be safe [8].

Anaphylaxis can present similarly to bronchospasm. This is divided into an allergic and a nonallergic constellation of symptoms. The allergic pathway requires an initial allergen exposure (most commonly neuromuscular blocking agents and antibiotics) to trigger IgE antibodies to bind to mast cells and basophils, then on subsequent exposure, the allergen will cross-link these antibody-receptor units, resulting in the release of inflammatory mediators [5,9]. The resulting degranulation and release of inflammatory mediators result in the bronchoconstriction, capillary leak, and vasodilation that anaphylaxis is associated with and can be supported by elevated serum tryptase levels. Nonallergic reactions have been thought to originate from either complement activation (iodinated contrast), a direct nonspecific activation (vancomycin, propofol), or activation of Mas-related G protein-coupled X2 receptors [9]. Regardless of the mechanism, the result is similar to allergic anaphylaxis, though it may not always be associated with an elevated tryptase level; a normal tryptase does not exclude anaphylaxis [9]. 

While local policy should always be referenced during an airway emergency, multiple approaches can be utilised, some using the following mnemonics: "displacement of the ETT, obstruction of the ETT, patient factors (such as pneumothorax, pulmonary embolism, and others), equipment issues, and stacked breaths (DOPES); others suggesting a more “physical” approach consisting of issues with the ventilator, to the tube, and to the various anatomical components of the patient [1,3,10]. Regardless of the method chosen, immediate life-threatening causes should be considered and addressed swiftly to minimise hypoxia. Findings that may suggest a developing bronchospasm include wheeze or silence on auscultation, rise in inspiratory pressures, desaturations, and a change in the end-tidal CO2 waveform. Other factors, such as a rash or hypotension, are more common in an anaphylactic reaction but do not exclude bronchospasm from the differential [1]. Additionally, while bronchospasm, hypotension, and tachycardia are the most common initial signs in anaphylaxis, they are also very common isolated signs in the perioperative setting, further complicating accurate diagnosis [9]. 

Once bronchospasm is determined to be the likely causative factor, management should proceed to reverse the underlying pathophysiology in a multimodal form. Initial treatment will depend on whether the patient is ventilated or not and should consist of short-acting beta-2 agonists (salbutamol) via the tracheal tube/nebuliser/metered dose inhaler as appropriate, as well as long-acting muscarinic antagonists via similar pathways [11]. If the patient is ventilated, deepening the sedation with bronchodilatory agents such as ketamine or sevoflurane can help regain control and release the bronchoconstriction. IV magnesium can be given to aid bronchial smooth muscle relaxation by decreasing presynaptic movement of calcium and inhibiting acetylcholine release. Ketamine can also be considered with its effects on bronchodilation via N-methyl-D-aspartate (NMDA) receptors and decreased vagal airway constriction [11]. Steroids can be considered to reduce any histamine-mediated bronchoconstriction associated with the inflammatory pathway from mast cell, eosinophil, and CD4+ T lymphocyte activation [12]. 

## Conclusions

Bronchospasm is a pathology that we encounter with sufficient regularity to require recognising when it occurs and how to respond appropriately to ensure patient safety. While initially a broad differential must be considered, once bronchospasm has been identified, a rapid, multimodal approach should rapidly improve symptoms and vital signs, as well as maximise patient safety. Despite this, improvement may not always happen, and in those cases, an alternative diagnosis must be considered. Fortunately, the pathophysiology underlying bronchospasm follows well-understood pathways, and the pharmacology associated with its correction is foundational to the perioperative doctor. While there were multiple possible differentials in the above case, the diagnosis of recurrent bronchospasm remains the most likely differential, within the limitations of no further investigations. A broad differential was considered in the case, with resolution of her symptoms following bronchospasm management. Had that management been insufficient in correcting her deterioration, then one of the other pathologies mentioned above would have been more likely, and management would need to change to correct the alternate pathology.

## References

[1] Westhorpe RN, Ludbrook GL, Helps SC (2005). Crisis management during anaesthesia: bronchospasm. Qual Saf Health Care.

[2] Ebo DG, Clarke RC, Mertes PM, Platt PR, Sabato V, Sadleir PH (2019). Molecular mechanisms and pathophysiology of perioperative hypersensitivity and anaphylaxis: a narrative review. Br J Anaesth.

[3] Fosse K, Salomonsen M, Gisvold SE, Gundersen B, Nordseth T (2025). Can intubate, cannot ventilate: A proposed algorithm to handle problems with ventilation and oxygenation after intubation. Acta Anaesthesiol Scand.

[4] Looseley A (2026). Management of bronchospasm during general anaesthesia. https://resources.wfsahq.org/wp-content/uploads/uia27-Management-of-bronchospasm-during-general-anaesthesia.pdf.

[5] Harper NJ, Cook TM, Garcez T (2018). Anaesthesia, surgery, and life-threatening allergic reactions: epidemiology and clinical features of perioperative anaphylaxis in the 6th National Audit Project (NAP6). Br J Anaesth.

[6] Takazawa T (2026). Perioperative anaphylaxis in Japan: Epidemiology, diagnosis, and challenges. Allergol Int.

[7] Takazawa T, Horiuchi T, Nagumo K (2023). The Japanese epidemiologic study for perioperative anaphylaxis, a prospective nationwide study: allergen exposure, epidemiology, and diagnosis of anaphylaxis during general anaesthesia. Br J Anaesth.

[8] Yamakage M, Iwasaki S, Namiki A (2008). Guideline-oriented perioperative management of patients with bronchial asthma and chronic obstructive pulmonary disease. J Anesth.

[9] Ma M, Duncan D, Bartoszko J (2025). Perioperative anaphylaxis: an update on pathophysiology, diagnosis, and management. Can J Anaesth.

[10] Nickson C (2026). Post-intubation hypoxia. https://litfl.com/post-intubation-hypoxia/.

[11] Khara B, Tobias JD (2023). Perioperative care of the pediatric patient and an algorithm for the treatment of intraoperative bronchospasm. J Asthma Allergy.

[12] Ramsahai JM, Wark PA (2018). Appropriate use of oral corticosteroids for severe asthma. Med J Aust.

